# Optical Microcavity: Sensing down to Single Molecules and Atoms

**DOI:** 10.3390/s110201972

**Published:** 2011-02-07

**Authors:** Tomoyuki Yoshie, Lingling Tang, Shu-Yu Su

**Affiliations:** Electrical and Computer Engineering, Fitzpatrick Institute for Photonics, Duke University, Durham, NC 27708, USA; E-Mails: lingling.tang@alumni.duke.edu (L.T.); shuyu.su@duke.edu (S.-Y.S.)

**Keywords:** microcavity, index sensing, absorption sensing, quality factor, photonic crystal, whispering gallery mode, surface Bloch mode, single molecule, single atom, cavity QED

## Abstract

This review article discusses fundamentals of dielectric, low-loss, optical micro-resonator sensing, including figures of merit and a variety of microcavity designs, and future perspectives in microcavity-based optical sensing. Resonance frequency and quality (Q) factor are altered as a means of detecting a small system perturbation, resulting in realization of optical sensing of a small amount of sample materials, down to even single molecules. Sensitivity, Q factor, minimum detectable index change, noises (in sensor system components and microcavity system including environments), microcavity size, and mode volume are essential parameters to be considered for optical sensing applications. Whispering gallery mode, photonic crystal, and slot-type microcavities typically provide compact, high-quality optical resonance modes for optical sensing applications. Surface Bloch modes induced on photonic crystals are shown to be a promising candidate thanks to large field overlap with a sample and ultra-high-Q resonances. Quantum optics effects based on microcavity quantum electrodynamics (QED) would provide novel single-photo-level detection of even single atoms and molecules via detection of doublet vacuum Rabi splitting peaks in strong coupling.

## 1. Introduction

A narrow resonance spectrum and enhanced light-matter interaction are unique characteristics of a dielectric microcavity [1] that are fundamental parameters limiting the performance of microcavity optical sensing. Biochemical sensing based on optical microcavity [2–4] is enabled via detection of modified optical properties of the microcavity mode. On the other hand, surface plasmon polariton (SPP) modes are often used for sensing and spectroscopy applications including SPP sensors and surface enhanced Raman spectroscopy (SERS) [5–9], for which the intensified field at the surface interacts with particles, molecules or liquid, and induces resonance shift and strong Raman signals. However, we do not discuss optical sensing based on SPP [10–14]; we focus on dielectric microcavities for optical sensing in this article.

State-of-the-art optical microcavities can show sharp resonances limited by material absorption in dielectrics. For example, toroidal optical microcavities [15,16] are demonstrated to show resonance linewidth of less than 0.0155 pm at λ = 1,550 nm resonance vacuum wavelength, corresponding to quality (Q) factor of 10^8^. Light-matter interaction within the microcavity is enhanced owing to photon trapping effect. The photon decay time τ is as long as 82.2 nsec, corresponding to 1.59 × 10^7^ optical cycles, for Q factor of 10^8^ at 1,550 nm wavelength of light. One of fundamental characteristic values of microcavities is the Q factor defined by

(1) $$Q=\omega \frac{U}{P}=\omega \frac{U}{-\mathrm{dU}/\mathrm{dt}}$$

where *ω*, *U*, and *P* are resonance angular frequency, stored energy and power loss from the microcavity. The quality factor calculation is required to consider all kinds of modal power loss, including radiation, material absorption, and scattering losses. For exponentially decaying time dependence of stored energy or electric field function, that is *E* = *E*_0_ exp (−*t* / 2*τ*)*u*(*t*) where *u*(*t*) = {1:*t* ≥ 0,0 : t < 0}, we can derive the following relation via Fourier transform of electric field; *Q* = *ωτ* = *ω* / *δω* = *λ* / *δλ* where *δω* and *δλ* are the resonance linewidths in angular frequency and wavelength domains. This relation is convenient as we can measure the linewidth by a scanning monochromator or a tunable laser in laboratory. Opposed to the finesse, the Q factor does not require the measurement of neighboring modes in a spectrum, which is difficult for single-mode optical microcavities.

The enhanced light-matter interaction is observed through a resonance shift and the reduction of the resonator Q factor. The resonance shift is mainly caused by two reasons; local refractive index change and thermo-optic (TO) effect due to heat induced from light absorption. Even these small perturbations induce the resonance shift that is larger than the resonance linewidth.

Perturbations can also change the mode-to-mode coupling via light scattering. The detection of altered coupling enables optical sensing of small amounts of analytes [17,18]. Zhu *et al.* [17] measured the mode splitting of coupled modes that originated from clock-wise and counter-clock-wise whispering gallery modes (WGMs), and detected nanoparticles.

**Figure 1.**
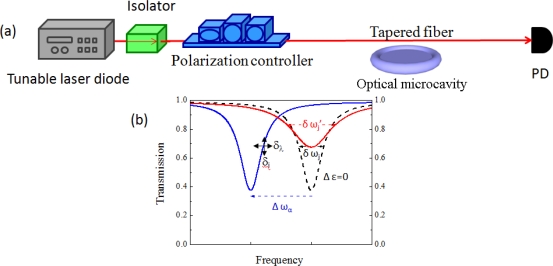
Microcavity optical sensing: **(a)** Optical sensing instrument example. Light from a tunable laser is coupled to a tapered fiber after isolator and polarization controller. Evanescent light on the tapered fiber is coupled to an optical microcavity mode. The transmittance signals are detected at a photodetector (PD). For practical sensing instrumentation, sensing system with microfluidics and optics can be integrated [21–24]. **(b)** Transmission spectra for index and Q-factor sensing.

Optical microcavities can perform label-free detection of single molecules due to the enhanced interaction of resonance light with molecules [15,19,20]. One typical microcavity-based optical sensing system is shown in Figure 1(a). Narrow laser spectrum line light with appropriate polarization is evanescently coupled to a microcavity via a tapered fiber. At resonance, the light is dropped into the optical resonator, and we see a dip in a transmission spectrum as seen in Figure 1(b); dashed black, blue, and red resonance dips represent ones without any perturbation, with index shift perturbation, and with absorptive perturbation. The values δω_j_ and δ ω_j_’ are full width at half maximum (FWHM) of resonances, and δ_i_ and δ_λ_ are intensity and wavelength noises, which depend on each system. Detection of the wavelength shift Δω_α_ or broadening δω_j_’−δω_j_ allows us to detect analytes on the microcavity.

**Figure 2.**
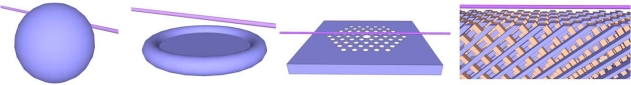
Representative microcavity geometries: Micro-sphere, micro-toroid, 2D photonic crystal slab, and 3D photonic crystal (woodpile) are shown from left to right. A tapered fiber proximate to each microcavity enables the evanescent field coupling to a microcavity mode. An integrated optical waveguide coupled to a microcavity is an alternative to the tapered fiber.

Representative microcavity designs are shown in Figure 2. Microcavity requires three-dimensional optical confinement, and high-reflectivity (HR) mirrors are often used. Metallic materials are not used due to absorption power loss. Instead, we use inhomogeneity of refractive index or simply index contrast. There are two major choices for obtaining high reflectance; total internal reflection (TIR) and distributed Bragg reflection. TIR demands that an angle of incidence on the interface is greater than the critical angle sin^−1^(n_1_/n_2_) for an interface of n_1_ and n_2_ media (n_1_ < n_2_), and TIR is expected. Combined TIR with a loop of optical trajectory, WGMs can be supported. The WGMs are essentially travelling wave resonator modes, and a modal optical round-trip satisfies constructive interference conditions. Representative WGMs include micro-sphere, micro-toroid, micro-disk, and micro-ring microcavity modes. On the other hand, distributed Bragg reflection is constructed based on destructive interference from multiple periodic scattering of light waves. Depending upon the dimension of distributed Bragg reflection, there are 1D, 2D and 3D photonic crystals; see Figure 2. Although it is possible to induce WGM-like modes in photonic crystals, the modes are typically constructed via standing waves.

Table 1 compares SPP, photonic and surface modes in terms of structure, sensitivity and Q factor. SPP and surface Bloch modes are surface waves. In order to support SPP modes, the negative value of the real part of the permittivity is required [25]. Therefore, the modes typically require metal. However, the SPP modes are lossy as indicated by ɛ_1_ = n’^2^ − k^2^ < 0 where the index of refraction is n = n’+ ik. The sensitivity S = dλ/dn is large thanks to large field penetration into air, but the mode resonance is spectrally broad due to large optical absorption, *i.e.*, quick photon decay time τ. We discuss photonic modes and surface Bloch modes in this article.

## 2. Resonance Perturbation

Resonance states are modified due to a system perturbation induced upon an event of analyte arrival at a microcavity. The modifications detected by the sensing system are typically either those of resonance wavelength or Q factor (*i.e.*, linewidth); we call each index shift or Q-factor sensing.

### 2.1. Resonance Frequency Shift

This resonance change can be expressed in terms of permittivity change by the perturbation theory [26]. We assume that the unperturbed, non-magnetic system defined by *ɛ*(*r⃗*) supports the mode *j* of resonance frequency *ω_j_* and modal electric field *E⃗_j_*(*r⃗*). Upon an arrival event of an analyte on the microcavity, the system is modified from *ɛ*(*r⃗*) to *ɛ*(*r⃗*) + Δ*ɛ*(*r⃗*). The resonance (angular) frequency shift due to a small perturbation in the permittivity Δ*ɛ*(*r⃗*) is given by

(2) $$\Delta \omega_{j}≅-\frac{\omega_{j}}{2}\frac{\int \Delta ɛ\left(\vec{r}\right){\left|{\vec{E}}_{j}\;\left(\vec{r}\right)\right|}^{2}d^{3}r}{\int ɛ\left(\vec{r}\right){\left|{\vec{E}}_{j}\;\left(\vec{r}\right)\right|}^{2}d^{3}r}$$

where Δ*ɛ*_1_(*r⃗*) = Re[Δ*ɛ*(*r⃗*)] and Δ*ɛ*_2_(*r⃗*) = Im[Δ*ɛ*(*r⃗*)].

The resonance shift is effective for the change in the real part of permittivity, *i.e.*, Δ*ɛ*_1_(*r⃗*). The index sensing detects the resonance wavelength shift shown in Equation (2). The resonance Q of the unperturbed system is equal to *ω_j_/δω_j_*. Assuming no change in the Q value, which is true for transparent molecules, we can sense the small perturbation when the resonance shift is comparable or exceeds the resonance linewidth *δω_j_* As seen in Equation (2), the analyte position must have a sufficiently high optical field of the mode *j*. The sensitivity is greatly dependent upon the field overlap of the analyte.

### 2.2. Q Factor Change

Monitoring the optical Q factor change, we can detect an analyte. This is so-called Q-factor sensing, typically based on absorption perturbation. Although many works use resonance shifts for sensing (*i.e.*, index sensing), the Q factor reduction can also be monitored for the detection of single molecules. Conventional non-surface-based microcavities lack sufficient light-matter interaction with single molecules. In this regard, surface-mode-based sensors would be advantageous. The Q factor can be strongly altered due to the small perturbation. Next, we consider probing the Q factor for sensing. The original unperturbed Q factor is limited by radiation and scattering loss on the microcavity or by absorption of microcavity dielectric media. As an increase in the perturbation strength, the Q factor [26] is decreased to

(3) $$Q\prime =\frac{Q-{\mathrm{QR}}_{1}/2-R_{2}/4}{1-R_{1}/2+{\mathrm{QR}}_{2}}$$

where

(4) $$R_{i}=\frac{\int \Delta {ɛ}_{i}\;\left(\vec{r}\right){\left|E_{j}\;\left(\vec{r}\right)\right|}^{2}d\vec{r}}{\int ɛ\left(\vec{r}\right){\left|E_{j}\;\left(\vec{r}\right)\right|}^{2}d\vec{r}},\left\{i=1,2\right\}.$$

Further increase in the absorption dominates the microcavity loss mechanism so that the Q factor drops further to

(5) $$Q\prime =\frac{Q}{1+{\mathrm{QR}}_{2}}\to \frac{1}{R_{2}}=\frac{\int ɛ\left(\vec{r}\right){\left|{\vec{E}}_{j}\;\left(\vec{r}\right)\right|}^{2}d^{3}r}{\int \text{Im}\left[\Delta ɛ\left(\vec{r}\right)\right]{\left|{\vec{E}}_{j}\;\left(\vec{r}\right)\right|}^{2}d^{3}r}.$$

The Q factor reduction is significant by increasing the light-matter interaction, *i.e.*, increasing the value of R_2_. The Q factor can be varied from Q to Q’ = R_2_^−1^ so that the dynamic range is QR_2_. Although the state-of-the-art microcavities are demonstrated to perform label-free, single-molecule detection, there are few kinds of single molecules that have been demonstrated to be detected. An absorptive analyte can be sensed via the Q factor sensing. This is unique in dielectric microcavity as SPP modes have a metal-absorption-limited, low-Q factor given by the Equation (5) without any analytes.

### 2.3. Sensitivity

The sensitivity is one figure of merit value for resonance shift optical microcavity sensing. It is typically defined by *S* ≡ *∂λ / ∂n* where *n* is the refractive index of the media containing analytes, and its typical unit is nm/RIU (refractive index unit: unitless). For transparent analytes, the sensitivity can be written from the Equation (2) as

(6) $$S=\frac{{ɛ}_{0}\lambda^{2}}{\pi c}\frac{\int_{\mathrm{env}}{\left|E_{j}\right|}^{2}d\vec{r}}{\int ɛ{\left|E_{j}\right|}^{2}d\vec{r}}$$

where the volume integral on the numerator runs over a volume containing the analytes. As the sensitivity has a unit of length, it is inconvenient to use the definition *S* ≡ *∂λ / ∂n* for comparisons among different sensing microcavity systems. Therefore, we introduce a dimensionless sensitivity normalized by the reference length *a*. It should be noted that the length *a* can be arbitrary in general or lattice spacing in photonic crystals. The normalized mode frequency is given by *f* = *a* / *λ*. The normalized sensitivity S’ is then written by

(7) $$S\prime \equiv -\frac{1}{f}\frac{\mathrm{df}}{\mathrm{dn}}=\frac{S}{\lambda }.$$

As seen in Equation (7), in general, long wavelength resonance has a large sensitivity value S. Comparisons among different microcavity systems become appropriate by the use of the normalized sensitivity S’. The normalized sensitivity is increased by optimizing field overlap with analytes so that it is desired to place the analytes on field maxima or large field points.

### 2.4. Minimum Detectable Index Change

**Figure 3.**
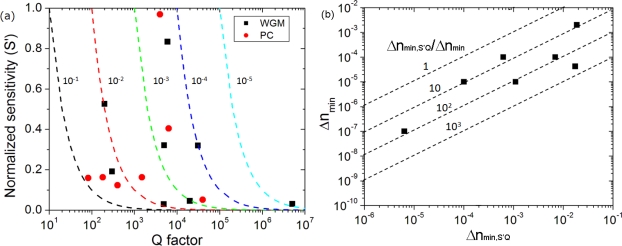
**(a)** Normalized sensitivity S’ *versus* Q factor. The data are collected from some papers on 1D photonic crystal nanobeam, 2D photonic crystal slab, microdisk, and micro-toroid optical resonators [27–40]. Dashed lines are contour lines of the value Δ*n*_min,*S*′*Q*_. **(b)** System minimum detectable index change Δ*n*_min_ *versus* the value Δ*n*_min,*S*′*Q*_ *=* (*S*′*Q*)^–1^. The dashed lines represent the improvement factor, *i.e.*, Δ*n*_min,*S*′*Q*_ / Δ*n*_min_.

The smallest detectable refractive index change by a sensor microcavity provides a good and practical figure of merit, but true minimum detectable index change in its total system may be dependent upon the measurement system including curve fitting method and the quality of light source and detector, *i.e.*, system noises. It would not be convenient to consider all kinds of noises and compare the quality of sensing microcavity. Therefore, we use the minimum detectable index change Δ*n*_min,*S*′*Q*_ at which the wavelength shift is equal to the linewidth of a resonance mode, *i.e.*,

(8) $$\Delta n_{\text{min},S\prime Q}=\frac{1}{S\prime Q},$$

which is independent of operation wavelength. Under an assumption of negligible noise, the minimum detectable index change can be reduced by the increase of the normalized sensitivity S’ and Q factor. Figure 3(a) shows the sensitivity, Q factor and Δ*n*_min,*S*′*Q*_ = (*S*′*Q*)^–1^ for data available from literature. The figure of merit given by Equation (8) easily compares sensing microcavities with different S’ and Q. Resonance curve fittings can significantly improve actual Δ*n*_min_ value with a factor of approximately 10 to 10^3^. This can be seen in Figure 3(b); this is also compiled from available data in literature showing the value S, Q and system minimum detectable index change.

### 2.5. Noises

Noises are often an important factor determining the minimum detectable index change. Hu *et al.* [41] theoretically studied the effects of two kinds of noises—intensity noise and wavelength noise—on the time-averaged sensitivity, which is an inverse of the index change Δ*n*_min_ that produces a signal to noise ratio of unity, normalized to square root unit equivalent bandwidth *df* / *N* as

(9) $$S^{*}=\sqrt{\frac{\mathrm{df}}{N}}\frac{1}{{\Delta n}_{\text{min}}}=\sqrt{\frac{\mathrm{df}}{N}}S\prime Q$$

where *df* ∼ 2*π* / *τ* (τ: integration time) and N discrete wavelength values for determining the resonance curve. Intensity and wavelength noises are treated independently. For a certain noise value in intensity and wavelength, the minimum detectable wavelength shift Δ*λ*_min_ is proportional to $1/\sqrt{Q}$ and $\sqrt{Q}$, respectively. Therefore, in order to minimize Δ*n*_min_ value, it is important to suppress the wavelength noise and use ultra-high-Q (UHQ) resonance.

Temperature fluctuations including those from thermo-optic effect are one source of wavelength noise. Other factors influencing wavelength noises result from other sensing instrument components such as accuracy and repeatability of wavelength in a tunable laser and a spectrometer.

### 2.6. Thermo-Optic Effect

The refractive index change due to the TO effect is given by *dn* = (*dn / dT*)*dT* where *dn / dT* is the thermo-optic coefficient; this is often treated as a linear term. The TO effect can act positively or negatively. When the temperature fluctuations are well suppressed, the resonance shift due to the TO effect can be utilized as a sensing scheme. Optical absorption by the system including analytes induces the temperature increase and thus refractive index shift. On the other hand, when the temperature fluctuation noises are large, the effect acts negatively. Depending upon sensing scheme, index drift due to the TO effect is enhanced or suppressed. Using the Equation (2), we find the resonance frequency shift due to the TO effect and normal index shift Δ*n_a_* as

(10) $$\Delta \omega ≅-\omega_{j}\frac{\int n\left(\vec{r}\right)\left\{\Delta n_{a}\left(\vec{r}\right)+\left(\frac{\partial n\left(\vec{r}\right)}{\partial T}\right)\Delta T\left(\vec{r}\right)\right\}{\left|{\vec{E}}_{j}\;\left(\vec{r}\right)\right|}^{2}d^{3}r}{\int n{\left(\vec{r}\right)}^{2}{\left|{\vec{E}}_{j}\;\left(\vec{r}\right)\right|}^{2}d^{3}r}$$

where Δ*n_a_*(*r⃗*) is the refractive index change due to analytes, *i.e.*, local index change. We can choose at least one of the following schemes in order to, for example, suppress the resonance shift due to the TO effect; (i) Total optical absorption must be suppressed while we maintain required input light power, so temperature increase is negligible. (ii) The sensing system including analytes is appropriately constructed so that the term $\int n\left(\vec{r}\right)\left(\partial n\left(\vec{r}\right)/\partial T\right)\Delta T\left(\vec{r}\right){\left|{\vec{E}}_{j}\left(\vec{r}\right)\right|}^{2}d^{3}r$ in the numerator of the Equation (10) is zero or sufficiently small. The TO coefficients (*dn / dT*) can be positive or negative [42]. An appropriate choice of materials is essential. Of course, we can design the system so that TO-effect-induced resonance shifts can be used. This case, we should still suppress temperature effect of the system but the analytes so that the temperature increases indeed come from the optical absorption by the analytes.

## 3. Optical Microcavity Designs for Optical Sensing

There are many optical microcavity sensing reports, and each microcavity design is unique in terms of Q factor, mode volume, size, and mode profile. In this section, we describe some examples of optical microcavities used in optical sensing technologies. As seen in Figure 3(a), there is more space to improve the figure of merit by Q factor enhancement than sensitivity, especially for dielectric optical resonators.

**Figure 4.**
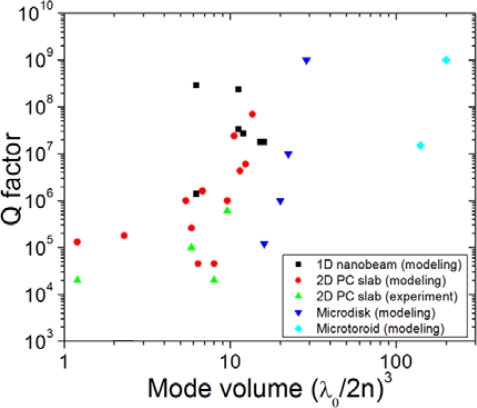
Q factor *versus* mode volume: The data are collected from some papers on 1D nanobeam, 2D photonic crystal slab, microdisk, and micro-toroid optical resonators [44–56].

For WGMs, the radiation loss degrading the Q factor is due to failure of TIR defined by an index contrast at an interface. WGM optical resonator geometries are well optimized for enhancement of Q factor, and micro-toroid resonators can show as large Q factor as 10^9^. Momentum component within an escape light cone results in radiation loss. In general, the passive (or unloaded) Q factor can be increased as an increase in the feedback size relative to a wavelength. Some Q factor enhancement cases are understood by far-field cancellation as, for example, presented by Vuckovic *et al.* [43]. For planar microcavities, the averaged radiated power is given by

(11) $$P=C\iint_{\left|\vec{k}\right|<k}dk^{2}\left({\left|H_{x}\;\left(\vec{k}\right)\right|}^{2}+{\left|H_{y}\;\left(\vec{k}\right)\right|}^{2}+\left({ɛ}_{0}/\mu_{0}\right){\left|E_{x}\;\left(\vec{k}\right)\right|}^{2}+\left({ɛ}_{0}/\mu_{0}\right){\left|E_{y}\;\left(\vec{k}\right)\right|}^{2}\right)$$

where the integration is performed within the line cone. As the mode number increases, the number of nodes increases and the cancellation increases. The Fourier function of an odd function is odd, and there is zero in the absolute square of the Fourier function, resulting in higher-order mode power cancellation. As a result, large mode number modes tend to yield large Q factor. Figure 4 shows the relation between Q factor and mode volume that are compiled from data available from some microcavity papers.

Field profile determines the sensitivity as seen in Equation (6). Opposed to surface modes, photonic dielectric modes tend to have large field localization in dielectrics, thus leading to low sensitivity. The larger the mode number is, the smaller sensitivity is likely. This is seen in Figure 3(a).

Mode volume is not an apparently important factor in figure of merit for conventional optical sensing in classical optics regime, but small mode volume tends to have a small detection volume so that it is useful for high-density sensing arrays. For a given analyte, the smaller the mode volume is, the larger the resonance shift is; this is understood from Equation (2). In this regard, small mode volume microcavities have an advantage. UHQ, small mode volume resonators are desired. On the other hand, small mode volume microcavities provide potentially novel optical sensing techniques based on quantum electrodynamics (QED) [57]. Although it is required to satisfy a strict condition, strong coupling between quantum electronic and photonic systems would provide ultimate optical sensing scheme as a single atom or molecule is detected by measuring optical response of the strong coupling system. The microcavity QED system [55,58–65] consists of a single atom (or molecule) and single mode filed, and its eigenmode becomes a doublet separated by 2g where g is the interaction rate. Without a molecule (*i.e.*, an analyte) we observe a resonator peak only in a spectrum. As a result, we can detect the atom or the molecule. In a strong-coupling cavity-QED system, large ratios $g/\kappa \propto Q/\sqrt{V}$ and $g/\gamma \propto 1/\sqrt{V}$ are desired, where κ and γ are decay rates of cavity and emitter. Thus, high Q and small V are required. Vacuum Rabi splitting was observed in various systems such as atom-photon and quantum dot-photon systems, meaning that the detection of a single atom or a single quantum dot was succeeded via measurements of doublet peaks. It should be noted that they are realized in low temperature and require precise positioning of single atoms

### 3.1. Whispering Gallery Mode Microcavity

**Figure 5.**
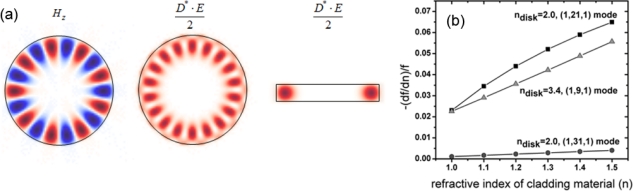
Microdisk resonator: **(a)** Mode profiles on cross-sections of a disk resonator. The H_z_ component and electric energy density D^*^ · *E* / 2 are shown for the (1,9,1) WGM. **(b)** Normalized sensitivity *S′* = −*f*^−1^(*df / dn*) *versus* refractive index of cladding media. WGM indices are (l,m,n) where l, m, and n are positive integers representing the order of modes along the radial, azimuth and z directions in the cylindrical coordinate system.

Total internal reflection along the circumference of a circular dielectric supports doubly-degenerate WGMs, which can be realized in several types of microcavities such as sphere [20,30,66–74], cylinder [75], disk [31,34,76], ring [28,77–80], toroid [15,17], and fiber coil [35,81] geometries. WGMs tend to provide large mode volume relative to photonic crystal ones, but the structure is compact and simple in fabrication. Figure 5 shows mode profiles of a microdisk and the normalized sensitivity S’ as a function of background index. Due to small penetration of field into low-index media, the sensitivity tends to be small. However, the mode number can be increased and this does not deteriorate the sensitivity significantly. Therefore, the factor S’Q can be high and the minimum detectable index change becomes small. Label-free single molecule detection events are reported via digital resonance shifts of UHQ WGMs [15,19,20].

### 3.2. Photonic Crystal Microcavity

Yablonovitch [82] and John [83] proposed to use 3D dielectric superlattice structures for studying suppression of spontaneous emission and amorphous 3D photonic crystal for strong localization in disordered dielectric superlattices, respectively. A photonic crystal is an artificial optical material based on sub-wavelength-scale interferences. Photonic crystals have either translational or rotational symmetry in permittivity and/or permeability with periodicity in order of wavelength. Efficient confinement of light in small volumes is a unique characteristic of photonic crystal microcavities. Two-dimensional photonic crystal slab microcavities have been the most popular choice among photonic crystals as the fabrication is simple and they provide large quality factor and small mode volume. One-dimensional photonic crystal nanobeam microcavity [84] also provides high-Q resonance and simple fabrication. A complete photonic bandgap is formed in a 3D photonic crystal, and omni-directional confinement of light is accomplished. With sufficiently large 3D photonic crystals [85–95], the Q factor is limited by dielectric absorption loss or scattering loss, not by radiation loss. For example, a monopole mode in woodpile has UHQ [95] and sufficiently large sensitivity (S’ ∼0.24 at n = 1.0) with small mode volume ∼ 0.4(λ/2)^3^.

**Figure 6.**
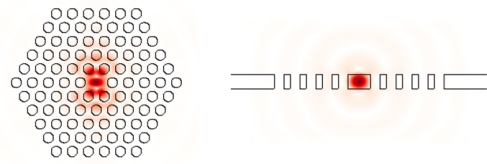
Mode profiles |E| of H1 2D photonic crystal microcavity.

The performance of two-dimensional photonic crystal slab microcavities has been advanced in the past decade, and there are many choices in terms of mode volume and Q factor. Popular 2D photonic crystal slab microcavity designs include H1 [96], L3 [97], and double-hetero [98] modes in a triangular lattice and D0 mode in a square lattice [99]; see Figure 6. For gas sensing, *i.e.*, background index ∼ 1.0, the calculated Q factors can exceed 1 million for mode volume of ∼ 2(λ/n)^3^. For background index ranging (1.3, 1.5), *i.e.*, liquid, the Q factor is reduced due to small index contrast; however it is still possible to obtain large Q factor.

### 3.3. Slot-Type Microcavity

**Figure 7.**
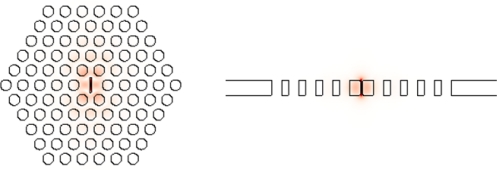
Mode profiles |E| of a slot microcavity in a defect in H1 2D slab photonic crystal. Q is 5.36 × 10^3^, and the mode volume is as small as 0.08(λ/2)^3^.

A discontinuity of permittivity enhances the electric field due to the continuity condition of the normal component of displacement vector at the discontinuity interface [100], *i.e.*, *E*_1⊥_ = (*ɛ*_2_ / *ɛ*_1_) *E*_2⊥_. The field can be enhanced by the factor of *ɛ*_2_ / *ɛ*_1_. As a consequence, the sensitivity is enhanced. Figure 7 shows a slot field enhancement in an H1 2D photonic crystal slab microcavity. Optical sensing with slot microcavity and waveguide [33,101–104] has been tested. They showed great potential of high-sensitivity optical sensing.

### 3.4. Microcavity Laser

Linewidth can be reduced by the introduction of gain [105], and thus reduction of Δ*n*_min_ is possible. One of the first microcavity laser sensing was done by Loncar *et al.* [22,106] who succeeded to show the possibility of femto-liter chemical detection and integration of the optical sensing with microfluidic platform. Kita *et al.* [107] recently showed chemical sensing by nanoslot laser that combined a photonic crystal slab microcavity with a permittivity discontinuity slot.

In general, microlasers tend to show much broader mode linewidth due to small photon numbers in the microcavity than large lasers. However, there are two advantages in microcavity laser: it does not require an external laser and the linewidth can be narrower than a passive microcavity with the similar mode volume size.

## 4. Surface Mode for Optical Sensing

**Figure 8.**
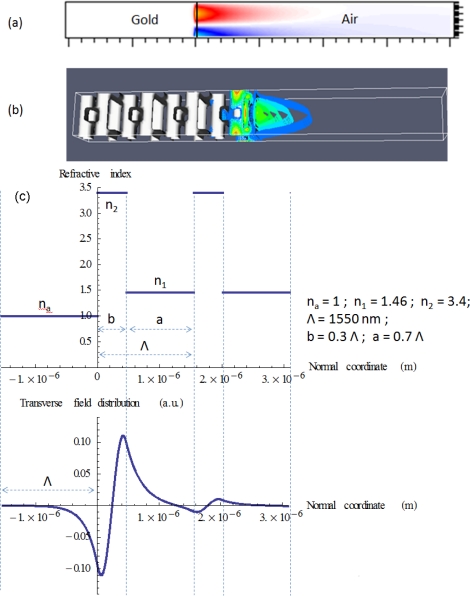
Mode profiles of surface modes: **(a)** Field penetration in air for SPP mode formed by air-gold interface. The H_y_ component is shown. The frequency is chosen to be close to cut-off so that field penetration is large in air. **(b)** Field profile |E| of surface Bloch mode induced on air/woodpile interface. The analysis results will be reported elsewhere. **(c)** Surface wave on a terminated 1D dielectric periodic structure.

Surface modes tend to have large sensitivity. SPP modes are induced in electromagnetic wave interaction with electrons in metal at surface, provided that the metallic media have negative real part of the permittivity. The fields exponentially decay from the interface and significantly overlap with air where we can place samples for detection; see Figure 8(a).

As seen in Figure 5 (WGMs) and Figure 6 (2D photonic crystal slab), for conventional photonic mode, most of the lightwave field and energy stay inside the microcavity. Although it is possible to slightly increase the field strength outside the microcavity, it is fundamentally difficult to enhance the field outside the microcavity significantly. On the other hand, surface modes [108–110] can utilize its large field on the surface for high-sensitivity optical detection of analytes. Conventional surface plasmon systems are lossy in optical frequency and it is difficult to build UHQ surface plasmon microcavities. There is a space to still improve the sensitivity by using UHQ surface microcavity modes.

The surface Bloch modes of photonic crystals are optical analogue to those of atomic crystals, and are excited in the lossless materials; see Figure 8(b). Yeh *et al.* [109] analyzed surface modes on a terminated 1D periodic dielectric structure; see Figure 8(c). Meade *et al.* [111] reported the first study of surface Bloch modes on a Yablonovite crystal, one of 3D dielectric photonic crystals. 2D photonic crystal slab surface modes are studied [32], and the surface modes are induced on the side facet of a membrane. 3D photonic crystal surface modes have a large surface area. The investigation of such surface Bloch modes has been hindered due to the lack of large 3D photonic crystals with complete photonic bandgap (PBG). Ishizaki *et al.* [88] experimentally confirmed surface modes on 3D photonic crystal. There still remain many tasks of applying the surface modes to sensing technology, but the work provides the possibility of high-sensitivity optical surface sensing. We analyzed surface Bloch modes on a terminated woodpile as seen in Figure 8(b). The sensitivity is high and the waveguide resonance is ultra-low-loss; the results will be reported elsewhere.

Fabrication of 3D photonic crystals is a challenge. Although many 3D photonic crystal designs show a complete PBG, the significance of woodpile 3D photonic crystals is that they are simple layered structures possessing a relatively large complete PBG. The woodpile photonic crystal is one of 〈100〉 diamond structured photonic crystals. Typical woodpile has approximately 15% complete PBG, defined by a complete PBG frequency width divided by a midgap frequency. The two-directional etching fabrication method [86,93,112] without wafer bonding can be used for woodpile photonic crystal fabrication. This is a promising fabrication technique which provides optical sensing on 3D photonic crystal with a complete photonic bandgap. Ultra-low radiation loss is possible due to the complete photonic bandgap.

## 5. Conclusions

Optical sensing technology by dielectric microcavity has been advanced significantly due to both demands of analysis of small amounts of analytes and rapid development of microcavity technology. This review article discusses fundamentals of optical micro-resonator sensing. Whispering gallery mode, photonic crystal, and slot-type microcavities typically provide compact, high-quality optical resonance modes for optical sensing applications. Surface Bloch modes induced on photonic crystals, are shown to be a promising candidate thanks to large field overlap with a sample and ultra-high-Q resonances. Quantum optics effects based on microcavity QED would offer a test bed for investigating novel, single-photon-level optical sensing of a single atom or single molecule.

## Figures and Tables

**Table 1.** Comparisons of optical modes: Photonic modes have oscillatory feature in the mode profile whereas an envelope function of surface Bloch mode exponentially decays.

	SPP mode	Photonic mode	Surface Bloch mode
Structure	Metal-dielectric interface	Dielectrics	Dielectric photonic crystal
Sensitivity	High to Very High	Low	High
Q factor	Low (10–10^2^)	High	High

## References

[1] Vahala K.J. (2003). Optical microcavities. Nature.

[2] Lukosz W. (1995). Integrated optical chemical and direct biochemical sensors. Sens. Actuat. B.

[3] Vollmer F., Arnold S. (2008). Whispering-gallery-mode biosensing: Label-free detection down to single molecules. Nat. Methods.

[4] Weiss S.M., Rong G., Lawrie J.L. (2009). Current status and outlook for silicon-based optical biosensors. Phys. E.

[5] Homola J. (2006). Surface Plasmon Resonance Based Sensors.

[6] Fleischmann M., Hendra P.J., McQuillan A.J. (1974). Raman spectra of pyridine adsorbed at a silver electrode. Chem. Phys. Lett.

[7] Wabuyele M., Yan F., Vo-Dinh T. (2010). Plasmonics nanoprobes: Detection of single-nucleotide polymorphisms in the breast cancer BRCA1 gene. Anal. Bioanal. Chem.

[8] Yan F., Vo-Dinh T. (2007). Surface-enhanced Raman scattering detection of chemical and biological agents using a portable Raman integrated tunable sensor. Sens. Actuat. B.

[9] White I.M., Gohring J., Fan X. (2007). SERS-based detection in an optofluidic ring resonator platform. Opt. Express.

[10] Chen H.M., Pang L., Kher A., Fainman Y. (2009). Three-dimensional composite metallodielectric nanostructure for enhanced surface plasmon resonance sensing. Appl. Phys. Lett.

[11] Hwang G.M., Pang L., Mullen E.H., Fainman Y. (2008). Plasmonic Sensing of biological analytes through nanoholes. IEEE Sens. J.

[12] Cooper M.A. (2003). Label-free screening of bio-molecular interactions. Anal. Bioanal. Chem.

[13] Gelfand R.M., Bruderer L., Mohseni H. (2009). Nanocavity plasmonic device for ultrabroadband single molecule sensing. Opt. Lett.

[14] Nakatani K., Sando S., Saito I. (2001). Scanning of guanine-guanine mismatches in DNA by synthetic ligands using surface plasmon resonance. Nat. Biotechnol.

[15] Armani A.M., Kulkarni R.P., Fraser S.E., Flagan R.C., Vahala K.J. (2007). Label-free, single-molecule detection with optical microcavities. Science.

[16] Zhang X., Choi H.S., Armani A.M. (2010). Ultimate quality factor of silica microtoroid resonant cavities. Appl. Phys. Lett.

[17] Zhu J., Ozdemir S.K., Xiao Y.-F., Li L., He L., Chen D.-R., Yang L. (2010). On-chip single nanoparticle detection and sizing by mode splitting in an ultrahigh-Q microresonator. Nat. Photonics.

[18] Koch B., Yi Y.S., Zhang J.Y., Znameroski S., Smith T. (2009). Reflection-mode sensing using optical microresonators. Appl. Phys. Lett.

[19] Arnold S., Shopova S.I., Holler S. (2010). Whispering gallery mode bio-sensor for label-free detection of single molecules: Thermo-optic *vs*. reactive mechanism. Opt. Express.

[20] Vollmer F., Arnold S., Keng D. (2008). Single virus detection from the reactive shift of a whispering-gallery mode. Proc. Natl. Acad. Sci. USA.

[21] Luan L., Evans R.D., Jokerst N.M., Fair R.B. (2008). Integrated optical sensor in a digital microfluidic platform. IEEE Sens. J.

[22] Adams M.L., Loncar M., Scherer A., Qiu Y.M. (2005). Microfluidic integration of porous photonic crystal nanolasers for chemical sensing. IEEE J. Sel. Areas Commun.

[23] Monat C., Domachuk P., Eggleton B.J. (2007). Integrated optofluidics: A new river of light. Nat. Photonics.

[24] White I.M., Oveys H., Fan X., Smith T.L., Zhang J. (2006). Integrated multiplexed biosensors based on liquid core optical ring resonators and antiresonant reflecting optical waveguides. Appl. Phys. Lett.

[25] Sernelius B.E. (2001). Surface Modes in Physics.

[26] Senlik O., Tang L., Tor-Ngern P., Yoshie T. (2010). Optical microcavities clad by low-absorption electrode media. IEEE Photonic. J.

[27] Chow E., Grot A., Mirkarimi L.W., Sigalas M., Girolami G. (2004). Ultracompact biochemical sensor built with two-dimensional photoniccrystal microcavity. Opt. Lett.

[28] De Vos K., Bartolozzi I., Schacht E., Bienstman P., Baets R. (2007). Silicon-on-Insulator microring resonator forsensitive and label-free biosensing. Opt. Express.

[29] DeLouise L.A., Kou P.M., Miller B.L. (2005). Cross-correlation of optical microcavity biosensor response with immobilized enzyme activity. Insights into biosensor sensitivity. Anal. Chem.

[30] Hanumegowda N.M., Stica C.J., Patel B.C., White I., Fan X. (2005). Refractometric sensors based on microsphere resonators. Appl. Phys. Lett.

[31] Krioukov E., Klunder D.J.W., Driessen A., Greve J., Otto C. (2002). Sensor based on an integrated optical microcavity. Opt. Lett.

[32] Lu T.W., Hsiao Y.H., Ho W.D., Lee P.T. (2009). Photonic crystal heteroslab-edge microcavity with high quality factor surface mode for index sensing. Appl. Phys. Lett.

[33] Robinson J.T., Chen L., Lipson M. (2008). On-chip gas detection in silicon optical microcavities. Opt. Express.

[34] Schweinsberg A., Hocde S., Lepeshkin N.N., Boyd R.W., Chase C., Fajardo J.E. (2007). An environmental sensor based on an integrated optical whispering gallery mode disk resonator. Sens. Actuat. B.

[35] Xu F., Horak P., Brambilla G. (2007). Optical microfiber coil resonator refractometric sensor. Opt. Express.

[36] Dündar M.A., Ryckebosch E.C.I., Nötzel R., Karouta F., van IJzendoorn L.J., van der Heijden R.W. (2010). Sensitivities of InGaAsP photonic crystal membrane nanocavities to hole refractive index. Opt. Express.

[37] Falco A.D., O’Faolain L., Krauss T.F. (2009). Chemical sensing in slotted photonic crystal heterostructure cavities. Appl. Phys. Lett.

[38] Levi O., Lee M.M., Zhang J., Lousse V., Brueck S.R.J., Fan S., Harris J.S. (2007). Sensitivity analysis of a photonic crystal structure for index-of-refraction sensing. Proc. SPIE.

[39] Ryckebosch E.C.I., Dündar M.A., Nötzel R., Karouta F., van IJzendoorn L.J., van der Heijden R.W. Refractive index sensing with an InGaAsP photonic crystal membrane cavity by means of photoluminescence.

[40] Sunner T., Stichel T., Kwon S.-H., Schlereth T.W., Hofling S., Kamp M., Forchel A. (2008). Photonic crystal cavity based gas sensor. Appl. Phys. Lett.

[41] Hu J., Sun X., Agarwal A., Kimerling L.C. (2009). Design guidelines for optical resonator biochemical sensors. J. Opt. Soc. Am. B.

[42] Palik E.D. (1998). Handbook of Optical Constants of Solids.

[43] Vuckovic J., Loncar M., Mabuchi H., Scherer A. (2002). Optimization of the Q factor in photonic crystal microcavities. IEEE J. Quantum Electron.

[44] Akahane Y., Asano T., Song B.-S., Noda S. (2005). Fine-tuned high-Q photonic-crystal nanocavity. Opt. Express.

[45] Englund D., Fushman I., Vuckovic J. (2005). General recipe for designing photonic crystal cavities. Opt. Express.

[46] Kuramochi E., Notomi M., Mitsugi S., Shinya A., Tanabe T., Watanabe T. (2006). Ultrahigh-Q photonic crystal nanocavities realized by the local width modulation of a line defect. Appl. Phys. Lett.

[47] Kuramochi E., Taniyama H., Tanabe T., Kawasaki K., Roh Y.-G., Notomi M. (2010). Ultrahigh-Q one-dimensional photonic crystal nanocavities with modulated mode-gap barriers on SiO_2_ claddings and on air claddings. Opt. Express.

[48] Loncar M., Hochberg M., Scherer A., Qiu Y. (2004). High quality factors and room-temperature lasing in a modified single-defect photonic crystal cavity. Opt. Lett.

[49] McCutcheon M.W., Loncar M. (2008). Design of a silicon nitride photonic crystal nanocavity with a Quality factor of one million for coupling to a diamond nanocrystal. Opt. Express.

[50] Nozaki K., Kita S., Baba T. (2007). Room temperature continuous wave operation and controlled spontaneous emission in ultrasmall photonic crystal nanolaser. Opt. Express.

[51] Song B.-S., Noda S., Asano T., Akahane Y. (2005). Ultra-high-Q photonic double-heterostructure nanocavity. Nat. Mater.

[52] Spillane S.M., Kippenberg T.J., Vahala K.J., Goh K.W., Wilcut E., Kimble H.J. (2005). Ultrahigh- Q toroidal microresonators for cavity quantum electrodynamics. Phys. Rev. A.

[53] Srinivasan K., Borselli M., Painter O., Stintz A., Krishna S. (2006). Cavity Q, mode volume, and lasing threshold in small diameter AlGaAs microdisks with embedded quantum dots. Opt. Express.

[54] Vuckovic J., Yamamoto Y. (2003). Photonic crystal microcavities for cavity quantum electrodynamics with a single quantum dot. Appl. Phys. Lett.

[55] Yoshie T., Scherer A., Hendrickson J., Khitrova G., Gibbs H.M., Rupper G., Ell C., Shchekin O.B., Deppe D.G. (2004). Vacuum Rabi splitting with a single quantum dot in a photonic crystal nanocavity. Nature.

[56] Zhang Z., Qiu M. (2004). Small-volume waveguide-section high Q microcavities in 2D photonic crystal slabs. Opt. Express.

[57] Mabuch H., Doherty A.C. (2002). Cavity quatnum electrodynamics: Coherence in context. Science.

[58] Houdre R., Gibernon J.L., Pellandini P., Stanley R.P., Oesterle U., Weisbuch C., Ogorman J., Roycroft B., Ilegems M. (1995). Saturation of the strong-coupling regime in a semiconductor microcavity—free-carrier bleaching of cavity polaritons. Phys. Rev. B.

[59] Peter E., Senellart P., Martrou D., Lemaitre A., Hours J., Gerard J.M., Bloch J. (2005). Exciton-photon strong-coupling regime for a single quantum dot embedded in a microcavity. Phys. Rev. Lett.

[60] Reithmaier J.P., Sek G., Loffler A., Hofmann C., Kuhn S., Reitzenstein S., Keldysh L.V., Kulakovskii V.D., Reinecke T.L., Forchel A. (2004). Strong coupling in a single quantum dot-semiconductor microcavity system. Nature.

[61] Srinivasan K., Painter O. (2007). Linear and nonlinear optical spectroscopy of a strongly coupled microdisk-quantum dot system. Nature.

[62] Englund D., Faraon A., Fushman I., Stoltz N., Petroff P., Vuckovic J. (2007). Controlling cavity reflectivity with a single quantum dot. Nature.

[63] Thompson R.J., Rempe G., Kimble H.J. (1992). Observation of normal-mode splitting for an atom in an optical cavity. Phys. Rev. Lett.

[64] Boca A, Miller R., Birnbaum K.M., Boozer A.D., McKeever J., Kimble H.J. (2004). Observation of the vacuum Rabi spectrum for one trapped atom. Phys. Rev. Lett.

[65] Manuz P., Puppe T., Schuster I., Syassen N., Pinkkse P.W.H., Rempe G. (2005). Normal-mode spectroscopy of a single-bound-atom-cavity system. Phys. Rev. Lett.

[66] Ren H.-C., Vollmer F., Arnold S., Libchaber A. (2007). High-Q microsphere biosensor—analysis for adsorption of rodlike bacteria. Opt. Express.

[67] Vollmer F., Braun D., Libchaber A., Khoshsima M., Teraoka I., Arnold S. (2002). Protein detection by optical shift of a resonant microcavity. Appl. Phys. Lett.

[68] Noto M., Keng D., Teraoka I., Arnold S. (2007). Detection of protein orientation on the silica microsphere surface using transverse electric/transverse magnetic whispering gallery modes. Biophys. J.

[69] Teraoka I., Arnold S., Vollmer F. (2003). Perturbation approach to resonance shifts of whispering-gallery modes in a dielectric microsphere as a probe of a surrounding medium. J. Opt. Soc. Am. B.

[70] Topolancik J., Vollmer F. (2007). Photoinduced transformations in bacteriorhodopsin membrane monitored with optical microcavities. Biophys. J.

[71] Arnold S., Khoshsima M., Teraoka I., Holler S., Vollmer F. (2003). Shift of whispering-gallery modes in microspheres by protein adsorption. Opt. Lett.

[72] Arnold S., Ramjit R., Keng D., Kolchenko V., Teraoka I. (2008). MicroParticle photophysics illuminates viral bio-sensing. Faraday Discuss.

[73] Keng D., McAnanama S.R., Teraoka I., Arnold S. (2007). Resonance fluctuations of a whispering gallery mode biosensor by particles undergoing Brownian motion. Appl. Phys. Lett.

[74] Vollmer F., Arnold S., Braun D., Teraoka I., Libchaber A. (2003). Multiplexed DNA quantification by spectroscopic shift of two microsphere cavities. Biophys. J.

[75] Blair S., Chen Y. (2001). Resonant-enhanced evanescent-wave fluorescence biosensing with cylindrical optical cavities. Appl. Opt.

[76] Boyd R.W., Heebner J.E. (2001). Sensitive disk resonator photonic biosensor. Appl. Opt.

[77] Chao C.-Y., Guo L.J. (2003). Biochemical sensors based on polymer microrings with sharp asymmetrical resonance. Appl. Phys. Lett.

[78] Yalcin A., Popat K.C., Aldridge J.C., Desai T.A., Hryniewicz J., Chbouki N., Little B.E., Oliver K., Van V., Sai C., Gill D., Anthes-Washburn M., Unlu M.S., Goldberg B.B. (2006). Optical sensing of biomolecules using microring resonators. IEEE J. Sel. Top. Quantum Electron.

[79] Sumetsky M., Windeler R.S., Dulashko Y., Fan X. (2007). Optical liquid ring resonator sensor. Opt. Express.

[80] Chao C.-Y., Fung W., Guo L.J. (2006). Polymer microring resonators for biochemical sensing applications. IEEE J. Sel. Top. Quantum Electron.

[81] Sumetsky M. (2008). Basic elements for microfiber photonics: Micro/nanofibers and microfiber coil resonators. IEEE J. Lightwave Technol.

[82] Yablonovitch E. (1987). Inhibited spontaneous emission in solid-state physics and electronics. Phys. Rev. Lett.

[83] John S. (1987). Strong localization of photons in certain disordered dielectric superlattices. Phys. Rev. Lett.

[84] Deotare P.B., McCutcheon M.W., Frank I.W., Khan M., Loncar M. (2009). High quality factor photonic crystal nanobeam cavities. Appl. Phys. Lett.

[85] Aoki K., Miyazaki H.T., Hirayama H., Inoshita K., Baba T., Sakoda K., Shinya N., Aoyagi Y. (2003). Microassembly of semiconductor three-dimensional photonic crystals. Nat. Mater.

[86] Cheng C.C., Scherer A. (1995). Fabrication of photonic band-gap crystals. J. Vac. Sci. Technol. B.

[87] Ho K.M., Chan C.T., Soukoulis C.M. (1990). Existence of a photonic gap in periodic dielectric structures. Phys. Rev. Lett.

[88] Ishizaki K., Noda S. (2009). Manipulation of photons at the surface of three-dimensional photonic crystals. Nature.

[89] Noda S., Tomoda K., Yamamoto N., Chutinan A. (2000). Full Three-dimensional photonic bandgap crystals at near-infrared wavelengths. Science.

[90] Ogawa S., Imada M., Yoshimoto S., Okano M., Noda S. (2004). Control of light emission by 3D photonic crystals. Science.

[91] Qi M., Lidorikis E., Rakich P.T., Johnson S.G., Joannopoulos J.D., Ippen E.P., Smith H.I. (2004). A three-dimensional optical photonic crystal with designed point defects. Nature.

[92] Tang L., Yoshie T. (2010). High-Q hybrid 3D-2D slab-3D photonic crystal microcavity. Opt. Lett.

[93] Tang L., Yoshie T. (2010). Woodpile photonic crystal fabricated in GaAs by two-directional etching method. J. Vac. Sci. Technol. B.

[94] Tang L., Yoshie T. (2007). Ultra-high-Q three-dimensional photonic crystal nano-resonators. Opt. Express.

[95] Tang L., Yoshie T. (2009). Monopole woodpile photonic crystal modes for light-matter interaction and optical trapping. Opt. Express.

[96] Painter O., Vuckovic J., Scherer A. (1999). Defect modes of a two-dimensional photonic crystal in an optically thin dielectric slab. J. Opt. Soc. Am. B.

[97] Akahane Y., Asano T., Song B.S., Noda S. (2003). High-Q photonic nanocavity in a two-dimensional photonic crystal. Nature.

[98] Song B.S., Noda S., Asano T., Akahane Y. (2005). Ultra-high-Q photonic double-heterostructure nanocavity. Nat. Mater.

[99] Lu T.-W., Lin P.-T., Sio K.-U., Lee P.-T. (2010). Optical sensing of square lattice photonic crystal point-shifted nanocavity for protein adsorption detection. Appl. Phys. Lett.

[100] Almeida V.R., Xu Q.F., Barrios C.A., Lipson M. (2004). Guiding and confining light in void nanostructure. Opt. Lett.

[101] Baehr-Jones T., Hochberg M., Walker C., Scherer A. (2005). High-Q optical resonators in silicon-on-insulator-based slot waveguides. Appl. Phys. Lett.

[102] Gylfason K.B., Carlborg C.F., Kazmierczak A., Dortu F., Sohlstrom H., Vivien L., Barrios C.A., van der Wijngaart W., Stemme G. (2010). On-chip temperature compensation in an integrated slot-waveguide ring resonator refractive index sensor array. Opt. Express.

[103] Lee S., Eom S.C., Chang J.S., Huh C., Sung G.Y., Shin J.H. (2010). Label-free optical biosensing using a horizontal air-slot SiNx microdisk resonator. Opt. Express.

[104] Skivesen N., Têtu A., Kristensen M., Kjems J., Frandsen L.H., Borel P.I. (2007). Photonic-crystal waveguide biosensor. Opt. Express.

[105] Yariv A., Yeh P. (2007). Photonics.

[106] Loncar M., Scherer A., Qiu Y. (2003). Photonic crystal laser sources for chemical detection. Appl. Phys. Lett.

[107] Kita S., Hachuda S., Nozaki K., Baba T. (2010). Nanoslot laser. Appl. Phys. Lett.

[108] Rahachou A.I., Zozoulenko I.V. (2006). Waveguiding properties of surface states in photonic crystals. J. Opt. Soc. Am. B.

[109] Yeh P., Yariv A., Hong C.-S. (1977). Electromagnetic propagation in periodic stratified media. I. General theory. J. Opt. Soc. Am.

[110] Vlasov Y.A., Moll N., McNab S.J. (2004). Observation of surface states in a truncated photoniccrystal slab. Opt. Lett.

[111] Meade R.D., Brommer K.D., Rappe A.M., Joannopoulos J.D. (1991). Electromagnetic Bloch waves at the surface of a photonic crystal. Phys. Rev. B.

[112] Takahashi S., Okano M., Imada M., Noda S. (2006). Three-dimensional photonic crystals based on double-angled etching and wafer-fusion techniques. Appl. Phys. Lett.

