# Correction: Yoshie, T. *et al*. Optical Microcavity: Sensing down to Single Molecules and Atoms. *Sensors* 2011, *11*, 1972–1991

**DOI:** 10.3390/s110606493

**Published:** 2011-06-22

**Authors:** Tomoyuki Yoshie, Lingling Tang, Shu-Yu Su

**Affiliations:** Electrical and Computer Engineering, Fitzpatrick Institute for Photonics, Duke University, Durham, NC 27708, USA; E-Mails: lingling.tang@alumni.duke.edu (L.T.); shuyu.su@duke.edu (S.-Y.S.)

The coefficient of the expression of Equation (6) was not properly written. Equation (6) should be corrected as

$$S≃\lambda \frac{\int_{env}n{ɛ}_{0}|E_{j}{|}^{2}\vec{dr}}{\int ɛ|E_{j}{|}^{2}\vec{dr}}$$

in our paper published in *Sensors* in 2011 [1].
